# Gross human rights violations and reparation under international law: approaching rehabilitation as a form of reparation

**DOI:** 10.3402/ejpt.v4i0.17191

**Published:** 2013-05-08

**Authors:** Nora Sveaass

**Affiliations:** Department of Psychology, University of Oslo, Oslo, Norway

**Keywords:** Gross human rights violations, redress, rehabilitation, torture, reparation

## Abstract

The strengthening of international criminal law through an increased focus on the right to reparation and rehabilitation for victims of crimes against humanity represents an important challenge to health professionals, particularly to those in the field of trauma research and treatment. A brief outline of some developments in the field of international law and justice for victims of gross human rights violations is presented, with a focus on the right to reparation including the means for rehabilitation. The fulfillment of this right is a complex endeavor which raises many questions. The road to justice and reparation for those whose rights have been brutally violated is long and burdensome. The active presence of trauma-informed health professionals in this process is a priority. Some of the issues raised within the context of states’ obligations to provide and ensure redress and rehabilitation to those subjected to torture and gross human rights violations are discussed, and in particular how rehabilitation can be understood and responded to by health professionals.

Strengthening of the international system to ensure accountability for crimes against humanity and justice for victims involves a stronger focus on the right to reparation, including the means for rehabilitation after torture and other gross human rights violations.^1^ This increasing emphasis, especially when extended to the obligation of states to provide rehabilitative care and services to victims of torture, challenges health professionals on many levels. It involves awareness and knowledge about the rights of victims and engagement and presence during legal procedures. Last but not least, it involves active participation, not only in providing care and rehabilitative services, but also in ensuring that the rights contained in international treaties are in fact effectively fulfilled. The obligation to provide redress, and in particular psychosocial and health-related care, rests on a number of conditions, among which political will to provide redress, coupled with actual, available and accessible services, are essential.

A major challenge today is how the international community can ensure that the justice it seeks in fact represents a “just” justice to those who have experienced gross violations of human rights. Necessary attention must be paid to the rights and needs of those involved in the process as victims, witnesses, or other parties. What contributions can the field of traumatology make to secure justice and reparation for those affected in a way that takes into account what they have endured? There is a need to discuss what rehabilitation as a form of reparation means and how this can be dealt with in practice. These questions are discussed below with a focus on how the idea of rehabilitation as a form of reparation or redress has developed within international human rights treaties. How may it be understood and dealt with from the point of view of health professionals? What are the challenges involved in fulfilling this right to those who have survived serious human rights crimes?

## The international justice project

Demand for the rights of victims and survivors of crimes against humanity to justice, to truth and to various forms of reparation has in recent years become an issue of high priority. In particular the right to reparation for harm suffered as part of international justice has been established as a way in which persons exposed to gross human rights violations can be compensated for what happened to them (Ferstman, Goetz & Stephens, 2009). But first some words about justice.

The 35-year sentence of Duch, the former Khmer Rouge prison chief in July 2010 was described as a major step for international justice. There has been a strong public awareness and interest in the arrests of perpetrators of crimes against humanity during the war in the Former Yugoslavia, such as Slobodan Milosevic, Radovan Karadzic and Ratko Mladić, and their transfer to stand trial before the International Criminal Tribunal for the former Yugoslavia (ICTY). Attempts at bringing former or present dictators to court, for instance the late Augusto Pinochet from Chile, Charles Taylor from Sierra Leone who have been tried at the International Criminal Court (ICC), or Omar Bashir from Sudan, the first head of state to be charged by this same court, are usually followed with great public interest, in particular by the survivors of the human rights abuses committed (Victims’ Rights Working Group, 2010).

Achievements in international criminal law are reflected in the coming into force of the Rome Statute of the ICC in 2002 (UN 1999–2002). International tribunals to process crimes against humanity committed in Rwanda and in former Yugoslavia that came into being prior to the ICC bore clear messages that a new era with regard to fighting impunity and holding perpetrators to account had begun. Impunity, or the fact that perpetrators of crimes against humanity, genocide and war-crimes could be granted immunity or be exempted from punishment, has been one of the serious impediments to justice and new social order. The Rome statute envisions that those responsible for such crimes should be tried in domestic courts when possible, that is, where there are the necessary conditions combined with a willingness to carry out justice. If this is lacking, justice must be carried out by or in collaboration with the international community. Today, we see a number of ongoing tribunals and courts, based on different models. These include international initiatives, combined national and international courts, the so-called hybrid courts (such as the Extraordinary Chambers in the Courts of Cambodia), or finally as national or domestic legal proceedings, such as the trials now taking place in Argentina and Chile, more than 30 years after the crimes took place.

## Fighting impunity

Much of what happened in international criminal law during the 1990s can be understood in light of the strong and engaged campaign against impunity, in particular in Latin-America. The fight against impunity began during the era of the military dictatorships, and has not diminished following the adoption of amnesty laws for crimes against humanity. In Chile, Argentina, and Uruguay, psychologists, doctors, and others who worked with torture survivors and families of disappeared persons within the framework of human rights organizations have argued that impunity must be considered as a continued and ongoing form of torture. Impunity for those responsible for crimes against humanity was regarded as detrimental to any reconstruction of society and incompatible with the process of healing and moving on in life. Diana Kordon, Dario Lagos, and Lucilla Edelman from Argentina, and Paz Rojas, Elisabeth Lira, and Maria Castillo from Chile are among those who have stressed the importance of not leaving this battle to the legal field alone. The fight against amnesty laws should also be based on arguments from a psychological and trauma-informed perspective (Lira & Castillo, 1991; Kordon, Edelman, Lagos, Nicoletti, Kersner, & Groshaus, 1992; Kordon, Edelman, Lagos, & Kersner, 1995; Rojas, 1993). They are still engaged fulltime in the fight against impunity and for justice and reparation, for the survivors and families of the disappeared, and for assistance, treatment, and follow-up of people severely traumatized—some from more than 30 years ago (Kordon, Edelman, & Lagos, 2010; Rojas, 2009).

## The long road to justice

Given the scope of international justice and the establishment of universal jurisdiction for grave and heinous crimes such as torture, it is high time to ask whether there has been sufficient thought and consideration given to the survivors, the victims, the witnesses, and the family members involved. After all justice for them is what this is all about. Universal jurisdiction implies that there must be no safe haven for those responsible for crimes against humanity. There should be no safe hiding place. If apprehended, extradition and court procedures should commence where the crimes have been committed or, if this is not feasible, in the state where the person has been detained. The principle involved here is the obligation known as “aut dedere aut judicare”. Extradite or prosecute.

What about those whose lives have been changed by these crimes? How safe do they feel, years after peace accords are agreed upon, after dictators have stepped down or been ousted? Do they feel protected against ongoing threats from those responsible for the crimes? Ensuring protection and participation for victims is essential. When war criminals or torturers are detained, those subjected to the violations will often express strong reactions, such as relief but also fear and awe. Who may be out there to stop their story from being told in court? What will happen if they tell what happened and even if they do, will they be believed? Questions such as these very soon surge to the surface (Džumhur, 2012; Hamber, 2009; Victims’ Rights Working Group, 2010). Many of those who have been exposed to the violations still struggle with feelings of fear and lack of trust in the system (Kordon, Edelman, & Lagos, 2010).

These are not arguments against the need for a continued and strengthened system of justice to deal with these crimes. On the contrary, impunity must be considered as being not only against international law but also psychologically detrimental (Rojas, 2000, 2009). The call for justice belongs not only to the legal world. It is very strong in the hearts and minds of those who have suffered human rights violations (Kordon, Edelman, Lagos, & Kersner, 1995; Rojas, Espinoza, Urquieta, & Soto, 1998; Sveaass, 1994; Sveaass & Lavik, 2000). The challenge is to have a strong justice system that reflects the expectations and wishes of the victims (Stover, 2005). This must include a strong focus on survivors’ rights and support and assistance for witnesses, legal as well as psychosocial. Judges and prosecutors focus on their role as protectors of justice, not necessarily protectors of those whose experiences are at the heart of the matter (Sveaass & Sønneland, 2010).

## Dialogue between the field of trauma and the system of justice

All attempts to define and develop a victim-centered and victim-friendly approach are commendable, essential, and should be a matter of priority. Knowledge of victims’ experiences and expectations of justice in relation to human rights violations is limited. Based on some of the few studies that have been undertaken, it would seem many victims are disinclined to pursue justice due to a lack of trust and limited information as to how things function. (Džumhur, 2012; Hamber, 2009; Kordon, Edelman, & Lagos, 2010; Stover, 2005; Victims’ Support Working Group, 2010; Uganda Victims Foundation, 2011). Why does the security of the defendants seem so much better taken care of than safety of the victims? Why are they provided with privileges that victims are not entitled to? Why are all those charged not detained? These are common questions among victims.

The legal proceedings themselves often represent a heavy burden and may be experienced as retraumatizing events. Just being in the same room with the alleged perpetrator may be an ordeal, likewise the process of testifying and perhaps being questioned in ways that may be humiliating. It is about telling the untold stories, and perhaps not being believed in court. It can be about exposure to threats or other frightening events outside the courtroom, and then at the end the possibility of seeing the accused person acquitted or given a minimal sentence. These aspects of the justice system in cases of severe human rights violations may scare, demotivate, or break down what remains of resistance and hope. They must be taken into consideration as possible and serious impediments to any aspiration toward a just process. Psychological and trauma-related reactions in those who have lost or survived must be considered possible obstacles to obtaining the rights inherent in conventions and treaties, and in the right to redress. Thus, a dialogue between the two worlds—caretakers of justice and caretakers of trauma victims—is needed in the pursuit of justice for victims of human rights abuses. In addition to providing assistance through witness protection and victim support programs, psychologists and doctors have important roles to play related to assessment and documentation of consequences of torture, for instance through medico-legal and psychological reports and thereby the provision of evidence (UN General Assembly, Interim report, 2010). They must be aware of state obligations and victims’ rights and be part of a system that monitors and oversees implementation of rights, including the right to reparation and rehabilitation, meaning the assistance and care needed to restore function and independence, as part of a reparative scheme.

## On reparation and rehabilitation

The right to reparation and in particular to rehabilitation, in international law is complex and usually refers to the obligation of the state responsible for torture to provide this as part of the compensation to victims. But ensuring rehabilitation to victims of torture depends on many different premises related both to state obligations and to the receivers themselves. The willingness of the state to provide services and the criteria they set for access to such services are important if effective redress and rehabilitation is to happen. And there are issues concerning the persons directly involved: The level of confidence that the person has with respect to receiving rehabilitation services offered by the authorities, the question as to whether the person still lives in the state where violence has been committed and whether the necessary steps have been taken with regard to complaints, assessments and documentation. Then, there is the question of what is meant by rehabilitation services. Do these consist mainly of health services or do they include a larger spectrum of other often needed forms of assistance, such as training and education, housing, legal assistance, and the like?

Last but not least, how does rehabilitation based on a medical model aimed at providing medically informed care and assistance, correspond to the fact that torture is usually inflicted within a context of political violence and conflict, and should be understood and dealt with from this perspective? The limitations of the medical model of rehabilitation have been a contentious point of discussion among those working with victims of torture at a global level, as part of the provision of psychosocial services. Not only because of inherent limitations in the model, but, as argued, as it is directly misleading and politically wrong to approach political actions and abuse power with medical terminology (Becker, 1995; Summerfield, 1995). This touches upon the major issues involved in the ambitions and obligations related to redress including rehabilitation. That is, ways in which a state that has been responsible for atrocities and violations as an intentional and purposeful way of maintaining power are obliged to redress the injustice and provide a means for rehabilitation of the damages done. Redressing damage can never be done by health measures or other reparation measures alone. This brings us back to the discussion of the wide array of justice, including criminal justice and holding perpetrators responsible, public apologies, and other social and individual reparative actions.

While no oppressive or violating act can be understood or dealt with within a medical framework, it is beyond doubt that violations such as torture and other forms of ill-treatment may create conditions or consequences that require assistance from medical or health professionals. How these rights are dealt with, met, and monitored to ensure compliance in practice is a major issue that clearly also involves trauma-informed professionals. It is easy to understand that some people will not avail themselves of such services, out of fear but also owing to a deep lack of confidence—at least not in the state where violations have occurred.

## What is rehabilitation?

There are a number of definitions of rehabilitation and of what is understood by services which aims at rehabilitation (Redress, 2009). The recently adopted General Comment nr. 3 to article 14 of the Convention Against Torture, argues that rehabilitation “should be holistic and include medical and psychological care as well as legal and social services”. Furthermore rehabilitation “refers to the restoration of function or the acquisition of new skills required as a result of the changed circumstances of a victim in the aftermath of torture or ill-treatment. It seeks to enable the maximum possible self-sufficiency and function for the individual concerned, and may involve adjustments to the person's physical and social environment. Rehabilitation for victims should aim to restore, as far as possible, their independence, physical, mental, social and vocational ability; and full inclusion and participation in society” (UNCAT, General comment nr. 3, 2012). Based on this definition, the obligations of states to provide redress, and in particular, the means for rehabilitation, should be based on these principles and include the elements described in the GC3.^2^

## Rehabilitation in practice

When discussing how rehabilitation fits into the larger scope of reparation under international law, it is necessary to look into the comprehensive work of rehabilitation with victims of torture and other gross human rights violations that have been carried out for years in the real world, by non-governmental centers, organizations and networks, and to a lesser extent by the states themselves. Health professionals working at such centers and the networks with which these centers are affiliated, like the International Rehabilitation Council for Torture Victims (IRCT) or the International Society for Health and Human Rights (ISHHR) have closely followed the developments in international human rights law. They have been strong voices for the right to redress and the need for rehabilitation services for all victims of torture (IRCT, 2005; van Willigen, 1992), irrespective of whether it is related to the right to redress or a free-standing right to rehabilitation after torture. The discussion as to how to deal with the sequaele of torture, understood as socio-political and repressive actions by authoritarian regimes or part of armed conflicts, has been kept high on the agenda in many of the centers around the world helping survivors of torture or ill-treatment, be it within the state where the violations took place or to refugees seeking protection. It is outside the scope of this article to go in depth in relation to this important work, but the following sites will provide substantive information (www.irct.com, www.ishhr.com, www.hhri.org).

## Reparation under international law

Reparative measures are intended to acknowledge harm, as well as repair or compensate whether state or non-state actors acting under the color of law commit the violations. One understanding of the term reparation denotes a process in which a person tries to come to terms with what has happened and enter a process of healing. It can refer to something that takes place in the individual, a complex psychological process that may be endangered or supported, but never directed or managed by others. Conditions can be favorable and beneficial or they can be detrimental and destructive to the process, but the process as such goes on in the hands, mind, and heart of the person (Hamber, 2009). Even when reparation is understood as a lengthy and complex psychological process, it remains linked to the different stages of a process toward justice, if there is one. It is reasonable to believe that experiences in relation to justice, both good and bad, will have repercussions on the person's capacity to heal, come to terms with what happened and move on.

Nevertheless, the focus here is on forms of reparation and redress as the term used in *human rights law*. That is the way in which states compensate loss and suffering, including through such actions, such as public apologies and rehabilitation. The term reparation is about what states do to right the wrongs, either because they see the need to do this, or because they are required to do so as a result of judicial proceedings brought forth by those who have suffered damages. Examples are the payments provided by the government of Chile to people tortured under the Pinochet regime, and by the government of Argentina to persons affected by disappearances and torture during the dirty war. Post-WWII Germany has given compensation to Jews following the Holocaust, to workers in the factories during the Second World War and issued programs of reparation for Jews returning to Germany after the war. An example of claims for compensation which have never been met includes the long struggle for redress from the government of Japan to the victims of their military's sexual slavery during WWII, or “comfort women” mostly from Korea. These examples deal with acts of reparation in response to events that took place long back.

## Rehabilitation in international human rights law: a free-standing right?

The right to *rehabilitation* has been referred to in the Convention on the Rights of the Child (CRC), the Convention on Economic, Social and Cultural Rights (CESC), and not least in the Convention on the Rights of Persons with Disabilities (CRPD). The duty of states to provide some form of rehabilitation to defined groups or to persons with certain characteristics or experiences is referred to and this obligation goes beyond the general concept of right to health.

The right to rehabilitation after torture could in principle be regarded as a right to all persons subjected to torture, that is, without reference to the right to reparation. Being a torture victim or survivor, would in itself bestow the person with a right to rehabilitation. This would be considered a free-standing right to those exposed to torture *and* in need of rehabilitation services. Should this right be limited to the person's own national state, or should it be considered a right that could be exercised everywhere? Should victims of torture be entitled to rehabilitation, regardless of *where* they are and *who* tortured them (Redress, 2009, 2010). This is perhaps how many have interpreted or at least chosen to interpret international conventions. Namely that given such devastating experiences, it should be a universal duty to provide them with health care and reintegrative services, without considerations as to whether formal complaints or court decisions have been made, to who was responsible for the torture or where it happened. The argument that rehabilitation facilities for torture victims should be established in all countries seems based on such an understanding (UN General Assembly, Iterim report, 2010). This would mean that an Iraqi refugee coming to Switzerland should be entitled not only to general health care, but also be given the option of a fuller rehabilitation directly related to the health damage suffered. In most cases this would imply something beyond what would usually be considered basic and necessary health care. It could be a matter of complicated dental treatment, long-term physiotherapy and/or psychotherapy, surgery, etc. Rehabilitation seen in this way is thus related to the need and experiences of the tortured person, and not primarily as a state obligation to provide compensation and redress for damages.

It has been argued that in order to strengthen this free-standing right to rehabilitation for victims of torture and other gross human rights violations one could directly invoke the rights entailed in the Convention of Persons with Disabilities (Reilly, 2010). Many victims of torture may in fact be considered as persons with disabilities, given the serious psychological and physical problems they encounter. This is another issue for discussion in a separate paper.

There is a significant gap between the establishment of rights and their implementation. Moreover, defining the rights of victims does not imply that persons in their situation will necessarily accede, seek, or obtain these rights. This is true for most of the disabled people in the world, including in the countries that have ratified the disability convention and it is certainly true for most of those who have been exposed to torture.

## Rehabilitation as part of redress

A narrower understanding is the right to rehabilitation as part of a compensatory scheme. What are the obligations of states to repair damages to victims of torture and their families? Article 14 of the United Nation Convention Against Torture requires the state to ensure that a person who has been tortured obtains redress including the means for as full rehabilitation as is possible (UN, 1984). As promising as this may seem, the fact is that most persons exposed to torture will be in immediate need of care and rehabilitation and will not be in a position to address and submit formal claims for reparation. Rehabilitation as reparation is a complex issue representing major challenges—practically, clinically, and legally. Who has the right to rehabilitation and how, when and by whom should it be given? (Redress, 2001, 2009, 2010). To approach this issue, some central human rights documents on rehabilitation as reparation will be referred to.

## Rehabilitation as redress in human rights documents

The first and perhaps still the strongest formulation of the right to rehabilitation as part of redress is found in the above-mentioned article 14 of the Convention Against Torture (UN, 1984)

Each state party shall ensure in its legal system that the victims of an act of torture obtains redress and has an enforceable right to fair and adequate compensation including the means for as full rehabilitation as possible.

There must be legislation in place not only to provide for redress but also for it to be an enforceable right, including means for rehabilitation. The state must ensure that there are laws regulating this, but also that there must be a system in place to provide such assistance or means for such rehabilitation. The scope and obligations under this article are now further elaborated in the Committee Against Torture's newly adopted general comment no. 3 on article 14.

The International Covenant on Civil and Political Rights does not refer to rehabilitation as reparation, but the Human Rights Committee in its general comments (general comment no. 20 from 1992 and no. 31 from 2004) has defined rehabilitation as a form of reparation (Human Rights Committee, 1992/2004). General comment no. 20 states that amnesties are unacceptable, among other reasons, because they would “deprive individuals of the right to an effective remedy, including compensation and such full rehabilitation as may be possible”. The Committee notes that

Reparation can involve restitution, *rehabilitation* and measures of satisfaction, such as public apologies, public memorials, guarantees of non-repetition and changes in relevant laws and practices, as well as bringing to justice the perpetrators of human rights violations. (Human Rights Committee, 1992/2004)

The adoption by the General Assembly in December 2005 of the *Basic Principles and Guidelines on the Right to a Remedy and Reparation for Victims of Gross Violations of International Human Rights Law and Serious Violations of International Humanitarian Law*, as a UN resolution was an important step in the process of strengthening international focus on the right to remedy and on *forms* of reparation (UN General Assembly, 2005; van Boven, 2010). The basic principles were the finalization of a long process and a lot of work, where Theo van Boven (1997), former UN Special Rapporteur on Torture and Other forms of Cruel, Inhuman and Degrading Treatment, and Cherif Bassiouni both played important roles. An early version of these principles (often known as the van Boven principles) established that rehabilitation shall be provided to include legal, medical, psychological, and other care and services as well as measures to “restore the dignity and the reputation of the victims”. Economic compensation was also referred to as a way in which medical and other expenses of rehabilitation can be obtained (van Boven, 1993). This resolution further states that:

In accordance with domestic law and international law, and taking account of individual circumstances, victims of gross violations of international human rights law and serious violations of international humanitarian law should, as appropriate and proportional to the gravity of the violation and the circumstances of each case, be provided with full and effective reparation, as laid out in principles 19 to 23, which include the following forms: restitution, compensation, *rehabilitation*, satisfaction and guarantees of non-repetition.

*Rehabilitation* should include medical and psychological care as well as legal and social services. It is also important to note that article 75 of the 1998 Statute of the ICC (the Rome Statute) provides that “The Court shall establish principles relating to reparations to, or in respect of, victims, including restitution, compensation and *rehabilitation*” (UN, 1999–2002).

As early as 2004, a resolution adopted by the Commission of Human Rights (2004/41) referred directly to the need for developing rehabilitation services, stressing that victims of torture “obtain redress and are awarded fair and adequate compensation and receive appropriate socio-medical rehabilitation” and encouraged “the development of rehabilitation centers for victims of torture”. In his report to the General Assembly in August 2010 (A/65/273) in his capacity as UN Special Rapporteur on Torture, Professor Manfred Nowak devoted a whole section to the role of rehabilitation centers for victims of torture, stating that it follows from the obligation of the Convention Against Torture that torture rehabilitation centers are established and that such centers must provide holistic treatment for survivors. He also makes an important point about the role rehabilitation centers have in providing evidence to hold perpetrators accountable. The active use of the Manual on Effective Investigation and Documentation of torture, the so-called Istanbul Protocol (2004) is important both to substantiate complaints of torture and to provide documentations that may be important in a context of justice and reparation.

## Redress and the UN Committee Against Torture

The resolutions and reports referred to are important as soft-law codification of the right of victims to remedy under international law, and in particular to a focus on rehabilitation as part of this. They do not have the same binding force as do the conventions, which are legally binding to the states that have ratified them. It is essential to go back to the Convention Against Torture, and explore how it applies to the issues of redress and rehabilitation.

The convention is today ratified by 153 states. On a regular basis, the states are asked to explain and document how the provisions of the convention are complied with. The provisions include the absolute prohibition against torture, the obligation to prevent torture and ill-treatment, including the obligation also to prevent and protect victims from gender-based violence, such as rape, domestic violence, female genital mutilation, and trafficking. Furthermore, states must report on how they meet their obligation to provide a victim of torture with redress, including the means for as full rehabilitation as possible. They are questioned on what is done to redress victims, what kind of rehabilitation is offered and must also provide statistics on this. States are frequently asked about training for medical personnel in detecting signs of torture, whether it includes training in the Istanbul protocol, and about the availability of health professionals to provide such services.

Typical questions posed to states would be for “information on any existing rehabilitation programs for torture victims and on any other steps taken by the State party to ensure medical and psychological rehabilitation” *(Jordan, May 2010, CAT/C/SR.932*: 45)^3^ and on “details on the measures taken by the State party to ensure rehabilitation, including psychological, for victims of acts of torture” (*Philippines, May 2009, CAT/C/SR.868*: 56). Similarly, asking the state party whether it “makes physical, psychological, and social rehabilitation services available to victims of torture or cruel, inhuman, or degrading treatment” (*Belgium*, 2008*, CAT/C/BEL/Q/2*: 31).

States may also be informed that

Reparation within the meaning of Article 14 has three dimensions: moral, financial, and medical. The payment of compensation alone is not enough; it is equally necessary to ensure victims the means necessary for their rehabilitation. It would be interesting to know in how many cases of torture the courts have ordered the payment of compensation to victims and whether there are rehabilitation programs in place (*Azerbaijan, May 2010, CAT/C/SR.909*: 35).

In the conclusions for Chad, it was pointed out that

The committee is deeply concerned about: (a) Persistent and consistent reports of torture and ill-treatment allegedly carried out by the State party's security forces and services, especially in district police stations, gendarmeries and remand centers, and the apparent impunity enjoyed by the perpetrators of such acts; … (d) Reports that torture and ill-treatment are commonly used on prisoners of war and political opponents. *The State party should: … (e) Offer full reparation, including fair and adequate compensation for the victims of such acts, and provide them with medical, psychological and social rehabilitation* (*Chad, May 2009, CAT/C/TCD/CO/1*: 17).

As can be seen, states are asked both about their legislation and about the actual existence of such services even in countries where most of the persons subjected to torture have arrived as refugees and where the state is not necessarily responsible for the torture. The Committee Against Torture is probably the treaty body that most frequently, most directly, and even most critically raises issues related to redress and rehabilitation. Despite most of the questions coming under the umbrella of rehabilitation as part of redress or compensation there are, as in the examples, also frequent references to services provided to torture victims. States can be asked about what kind of rehabilitative services they provide to traumatized refugees arriving in the country, or to victims of trafficking or others subjected to torture and ill-treatment, either within or outside the state. Are these services a form of reparation or a provision of necessary services to persons at risk? Again, it has been argued under the purview of universality both in civil and criminal jurisdiction, that one could actually be redressing violations committed by another country. Should there be established procedures permitting victims to recover reparation in countries other than where the actual torture took place (Hall, 2007)? This represents an important discussion on the scope of state obligations to provide redress, including rehabilitation, after torture.

## Ensuring rehabilitation

Rehabilitation to victims of torture depends on the clarification of some questions. First of all, the question of who has the right to redress after human rights violations must be addressed, as well as how to ensure that rehabilitation as part of redress is actually being carried out. Then, there is the question as to when and where rehabilitation can take place, by whom and what services will be available. These are questions about principles and definitions, but the questions are also closely linked to practical challenges on the ground and a political will to implement is what required.

## Who is entitled to rehabilitation and how can this be done?

It seems fair to say that all persons subjected to torture and ill-treatment and their dependents, in particular in cases of death or the disappearance of a person, should be entitled to redress, and with a possibility to access rehabilitation. This is a twofold obligation. At the procedural level, states must enact legislation and establish a complaint mechanism, investigation bodies, and institutions capable of determining the right to and awarding redress for a victim of torture and ill-treatment. At the substantive level, states must ensure that a victim of torture or ill-treatment obtains full and effective redress and reparative measures, including compensation and the means for as full rehabilitation as possible. Rehabilitation is a substantive right. Health professionals must be active in the process of ensuring that it is realized. Effective rehabilitation also rests on formal decisions that must be taken before rehabilitation as a form of redress can be provided. Must compensation be based on decisions taken by a court, and as such be delayed until liability has been established, or are other venues to redress possible. Must a perpetrator be identified, found guilty, and even convicted in court for the crime of torture before redress, including rehabilitation, can take place?

Some states will argue that the right to compensation can only be realized through a court order if a court recognizes the right of a victim of torture to compensation from the State, the victim will received compensation, including restitution if rights, adequate and equitable financial remedies, medical care and rehabilitation (UN, 2011: Kuwait, Periodic report to CAT, part 71, p. 13, May 2011).

Other states do not set forth the claim about court decisions and may have alternative ways of deciding upon the right to compensation, such as civil procedures, and through administrative measures.

These alternative ways of obtaining redress are very important, because after all, how many victims of torture will ever make it to court. Second, even if they did, how often do courts order rehabilitation as a measure of compensation? Standing up for your rights, and presenting complaints and stories about torture to the authorities in the country where torture has taken place is not only often dangerous, but something most people will have serious difficulties in doing so. For the many who have worked clinically with torture survivors, this is quite evident. Torture will very often reduce the capacity and the energy to deal with this part of the trauma, and most people will need a good psychosocial support system and one that they can be confident of, to want to raise such cases in the first place. The most valuable evaluation of good health and psychosocial support for survivors of torture may well be readiness to go to court when the treatment is over. Psychosocial and medical care is possibly a prerequisite for complaints and redress rather than a result of it.

Persons subjected to serious human rights violations have many reasons not to believe in the legal system in the state where the violations have taken place in addition to the lack of money, support, or self-confidence needed to raise one's own rights in a judicial setting. Legal aid is more often than not unavailable or often involves a very lengthy and complicated procedure. The wheels of justice turn slowly, with a high risk of retraumatization and lack of significant outcomes. Redress should not be dependent on the victim taking his or her case to court. The lack of available effective legal mechanisms, or the person's own choice *not* to pursue legal justice, should not prevent anyone from being able to access their right to redress through other means. The important challenge is to ensure that complex legal procedures do not stand in the way.

## When can rehabilitation take place?

Time is of the essence in ensuring the right to redress and rehabilitation. Judicial proceedings are generally lengthy, yet effective rehabilitation should begin at the earliest stage possible. It is detrimental if rehabilitation must be based on judicial or other lengthy and thorny processes, and this emphasizes the need for establishing alternative, non-legal channels. Health professionals with insight into trauma and the consequences of trauma should be engaged with the right to redress and reparation outside the prosecution context. Human rights abuses have a very special nature, with violations of a pervasive character and extreme humiliations often linked to feelings of shame. Given this, the seeking of reparation must be made possible through confidential procedures and with support from independent persons, such as health personnel. Recounting the trauma, especially in situations that may seem unsafe or not sufficiently trust-worthy may be retraumatizing and have detrimental effects. The possibility to actually obtain reparation thus depends on the implementation of practical, accessible, and confidential mechanisms. When this is not in place there is neither justice nor reparation to the victims, despite these being defined rights. The claim for justice and reparation may remain good ideas in an ideal world, instead of real rights on the ground. Trauma experts may be the necessary link between ideals and implementation.

## Where can it happen?

Whether redress is interpreted as a local or an international obligation, and whether one presumes civil or legal universal jurisdiction is important here. Many will wrongly argue that article 14 in the Convention Against Torture clearly specifies the obligation of the state that is responsible for the violations to provide redress and means to rehabilitation, and that this should be done in the state where the violations have taken place. All necessary rehabilitative services must be carried out in the state, or at least provided for by the state, by means of economic compensation. If it is understood under the auspices of international civil jurisdiction, redress can in fact be obtained in another state, and through this also rehabilitation. This would mean that the rehabilitation provided would then not only be regarded as health services but also as part of a redress scheme and as such as part of a universal obligation to redress crimes committed abroad (Hall, 2007).

## Who can do the work?

The Convention Against Torture defines that there is a state obligation to ensure that the person obtains redress and has an enforceable right to fair and adequate compensation including the means for as full rehabilitation as possible. It is important that it does not say enforceable right to rehabilitation but the means for as full rehabilitation as possible. This creates several possibilities such as determination to seek rehabilitation where the person decides, and not necessarily as directed and implemented by the state itself.

For those receiving rehabilitation this is highly relevant, because for the survivor, it is possible that not *any* doctor or health professional in *any* hospital would be acceptable as care-providers. Doctors at hospitals in postconflict states may have been involved in severe human rights violations years ago, for instance by falsifying certificates of death or birth, by refusing to assess and document signs of torture, or in other ways assisting the infliction of pain. Overcoming the effects of torture and other severe human rights violations is totally dependent on help not only being acceptable to the victim, but also part of a process where their wishes and opinions are valued. Victim participation is required and the professionalism and understanding of the care-providers must allow for a confident and safe environment. All of this represents *sine qua non* requirements of any rehabilitation. Based on this, it seems highly relevant to discuss the possibility that rehabilitation as reparation could be carried out by trauma experts working in national or international non-governmental organizations (NGOs), and that the state, through financing such services, actually fulfill their obligation to provide redress. This would mean that NGOs in Chile, Argentina, or the Philippines could use their expertise and provide rehabilitative services mandated and financed by the state. The state would then implement its obligations to provide means to rehabilitation and redress. This discussion is far from unproblematic, but it should be encouraged and possible ways in which to move in relation to this should be looked for.

## Future directions

There is a need for clarification on a number of issues related to the right to redress and in particular, in relation to the substantiation and implementation of these rights. One way to clarify, specify, and strengthen provisions and state obligations under article 14 of the Convention against Torture has been to develop and adopt a *general comment* on this article, as referred to above. The working document was reviewed by state parties, by NGOs, and by others engaged in victims’ rights. The general comment refers to many of the important conditions surrounding reparation in the form of rehabilitation and focuses on the importance of victim participation (UNCAT, general comment no. 3, 2012). It is my hope that the adoption of general comment no. 3, explaining and clarifying the obligations of state parties under article 14 of the UN Convention Against Torture and Other Cruel, Inhuman or Degrading Treatment or Punishment, will be one more step toward justice and reparation for all victims of torture. It does not happen by itself, and health professionals also have important contributions to make here.

## Concluding reflections

This article has been motivated by many challenges involved in the international aspirations for justice for crimes against humanity, in particular in the challenges that the victims and survivors are facing and the possible contributions and participations of health professionals in this field. Reparation has been discussed as an important element of justice, with a special focus on how the right to rehabilitation can be understood in the context of justice and reparation. Rehabilitation to survivors of torture regarded as a free-standing right to all persons who have been subjected to torture and crimes against humanity and as a basic form of reparation or redress have been outlined and some implications discussed. The torture convention and the rights and obligations laid down in this, are crucial but there is a need for further elaboration and to implementation. Active participation of trauma-informed personnel could contribute to the actual fulfillment in practice of the right to redress and to as full rehabilitation as possible. Their involvement should also imply that this is carried out in a way that ensures participation by and respect for those who have been severely affected by violence. Finally, the road to justice is long, and the many battles that have been won in relation to “*no to impunity”* and *yes to accountability for perpetrators*, must not preclude or overshadow the many hardships involved in obtaining justice for the survivors and their familes. This progress toward justice does not necessarily seem beneficial or meaningful for healing, but the solution does not lie in *less* justice but in “better justice”. It lies in closer participation with survivors, stronger advocacy for sound and secure ways of implementing the rights referred to and active involvement of the knowledge and understanding gained within traumatology. After all, this is about severe trauma and life-long consequences. It is about lack of protection and insecurity, about possible aggravating and retraumatizing factors, but it is also about ways out of the trauma. Ways of regaining dignity and reconstruction.

## Footnotes

1 This term encompasses the concepts of ‘‘effective remedy’’ and ‘‘reparation’’. The measures required to redress violations under the Convention entail restitution, compensation, rehabilitation, satisfaction, and guarantees of non-repetition.

2 As a reminder to the reader, the Convention Against Torture refers to compensation, including means for rehabilitation. It does not directly provide for rehabilitation, but for the means to obtain this, a formulation which is quite valuable in fact.

3 The concluding observations by the Committee Against Torture can be found in full text on http://www2.ohchr.org/english/bodies/cat/sessions.htm.
